# Suicidal ideation and associated factors among school-going adolescents in rural Uganda

**DOI:** 10.1186/1471-244X-7-67

**Published:** 2007-11-23

**Authors:** Emmanuel Rudatsikira, Adamson S Muula, Seter Siziya, Jeremiahs Twa-Twa

**Affiliations:** 1 Departments of Biostatistics and Epidemiology and Global Health, Loma Linda University, School of Public Health, Loma Linda, California, USA; 2 Department of Community Health, University of Malawi, College of Medicine, Blantyre, Malawi; 3 Department of Community Medicine, University of Zambia, School of Medicine, Lusaka, Zambia; 4 Principal Medical Officer, Child Health Division, Ministry of Health, Kampala, Uganda

## Abstract

**Background:**

Mental health is a neglected area of health research and practice in most of sub-Saharan African countries where the largest burden of morbidity is from infectious diseases. This even occurs despite the fact that some mental health problems may arise from infectious diseases.

**Methods:**

We conducted secondary analysis of the Uganda Global School-Based Health Survey-2003 to obtain the prevalence of, and assess factors that may be associated with suicidal ideation among school-going adolescents in rural Uganda. Assessment of association was conducted through both bi-variate and multivariate logistic regression analysis.

**Results:**

Altogether 21.6% of the study participants, 21.3% males and 23.5% females had seriously considered committing suicide within the past 12 months. Loneliness, worry were positively associated with suicide ideation after adjusting for age, gender, smoking, drinking, and experience of having been bullied (OR = 1.59; 95% CI [1.12, 2.26] and OR = 1.19; 95% CI [1.12, 2.25]) respectively. Males were less likely to seriously consider committing suicide than females (OR = 0.70; 95% CI [0.50, 0.98]).

**Conclusion:**

Adolescent suicidal ideation is a major public health issue in rural Uganda. Measures aimed to prevent adolescent suicides in Uganda should incorporate our understanding of factors that are associated with suicide in rural Uganda such the gender disparity and the association observed with substance use.

## Background

Globally, it is estimated that there were 786,000 suicide deaths in 1990 [1]. These deaths were only slightly lower than the number of deaths (900,000) estimated for lung cancer in 1990 [2]. Yet lung cancer has received attention among health care professionals and the general public while suicide has largely not attracted that much attention. There is paucity of data on suicidal ideation, attempted suicide and suicide among adolescents in Africa. This dearth of information is likely to be multi-factorial in origin, including in part the global lack of adequate interest in mental health research in general and limited funding. Many of the health systems in southern Africa are engaged more in the prevention and treatment of communicable diseases and not mental health issues. Even in developed nations, suicide among children has been a neglected issue until recently [3,4]. In the few setting were studies have been conducted, suicidal thoughts or ideation has been described as. Among Viennese student for instance, 37.9 percent reported having ever had suicidal thoughts in their lives [5]. Weissman *et al.* have assessed the lifetime prevalence for suicide ideation from nine countries [6]. This ranged from from 2.09% (Beirut) to 18.51% (Christchurch, New Zealand).

Ovuga *et al.*[7] have reported on lifetime prevalence of suicidal ideation among first year students admitted to Makerere University, Uganda. Among this sample, lifetime prevalence was 56.0% among first years student in disciplines other than medicine while prevalence was only 8.9% among first year medical students. We are unaware of previous research on suicidal ideation among in-school adolescents in rural Uganda. Our study was aimed to assess the prevalence and associated factors of suicidal ideation among school going adolescents in rural Uganda. We believe information obtained may the design, implementation and evaluation of public health intervention aimed to prevent suicides in Uganda.

## Methods

Our study was a secondary analysis of existing data available from the Uganda Global School-Based Health Survey (GSHS) conducted in 2003. The GSHS was developed by the World Health Organization (WHO) in collaboration with United Nations' UNICEF, UNESCO, and UNAIDS with technical assistance from Centers for Diseases Control and Prevention (CDC, United States). The GSHS aims to provide data on health and social behaviours among school-going adolescent students.

Variables that were used for our analysis included socio-demographic characteristics, having considered suicide in the past 12 months, tobacco cigarette and cannabis smoking, alcohol use, having been worried so as to affect sleep and parental supervision. The questions on suicide, worry and loneliness were: During the past 12 months, did you ever seriously consider attempting suicide? During the past 12 months, how often have you been so worried about something that you could not sleep at night? During the past 12 months, have you felt lonely?

The GSHS uses a two-stage probability sampling technique, in which for the first stage, primary sampling units are schools which are selected with a probability proportional to their enrolment size. In the second step a systematic sample of classes in the selected school are obtained. All students in the selected classes are eligible to participate. A self-completed questionnaire is used.

For the rural Uganda survey, overall response rate was 67 percent. Data analysis was performed using SUDAAN software version 9.0.1 (Research Triangle Institute, Research Triangle Park, North Carolina, United States of America). Our analysis was based on compete case analysis as no imputation of missing observations was carried out. We obtained frequencies of attributes. A weighting factor was used in the analysis to obtain prevalence estimates so as to reflect the likelihood of sampling each student and to reduce bias by compensating for differing patterns of non response. The weight used for estimation is given by the following formula:

W = W1 * W2 * f1 * f2 * f3 * f4

where W1 = the inverse of the probability of selecting the school

W2 = the inverse of the probability of selecting the classroom within the school

f1 = a school-level non response adjustment factor calculated by school size category (small, medium, large)

f2 = a class-level non response adjustment factor calculated for each school

f3 = a student-level non response adjustment factor calculated by class

f4 = a post stratification adjustment factor calculated by grade.

We also conducted logistic regression analysis to estimate the association between suicidal thoughts and relevant predictor variables. We report unadjusted bi-variate analysis between suicidal thoughts and a predictor variable. We thereafter report results for adjusted odds ratios for the factors.

## Ethical considerations

A committee with members drawn from the Ministry of Education and Ministry of Health oversaw data collection. The study protocol was reviewed and approved by the National Council for Science and Technology of Uganda. Permission to conduct the study was granted by the Headmasters of the respective schools. All eligible students were requested to complete an anonymous questionnaire. However, students were free to participate or not to participate. Non participation could either be overt, such as clearly indicating that they would not like to participate or by submitting a non-completed questionnaire.

## Results

Table 1 presents selected characteristics of the study population of 1,506 Ugandan school going adolescents aged 11 to 17 years (median15 years old) who participated in the study. Most of the sample was males (53.3%) and 16–17 years old (40.4%). The majority reported that parents supervised them when out of school (63.3%). One in 2 respondents had been bullied at least once in the last 30 days (52.3%), 5.7% were smokers, 17.7% were alcohol drinkers. 21.6% of the study participants, 21.3% males and 23.5% females had seriously considered committing suicide within the past 12 months.

**Table 1:** Socio-demographic and behavioral characteristics of study participants in the Global School Health Sample for rural Uganda, 2003

	Total N = 1,506	Males N = 784	Females N = 676
	% (n)	% (n)	% (n)

Age (years)			
11–13	13.4 (192)	10.2 (86)	14.3 (97)
14	21.0 (301)	15.9 (126)	24.0 (168)
15	29.4 (422)	28.6 (227)	26.7 (181)
16–17	36.2 (519)	45.2 (345)	35.0 (230)
Gender			
Females	46.3 (676)	-	-
Males	53.7 (784)	-	-
Worry			
No	54.5 (808)	25.1 (200)	24.3 (163)
Yes	45.6 (676)	74.9 (584)	75.7 (513)
Loneliness			
No	58.7 (857)	35.5 (264)	28.5 (190)
Yes	41.3 (603)	64.5 (520)	71.5 (486)
Cigarette smoking			
No	94.3 (1311)	92.1 (691)	96.8 (620)
Yes	5.7 (74)	7.9 (55)	3.2 (19)
Drinking			
No	82.3 (1071)	73.4 (553)	50.8 (518)
Yes	17.7 (230)	26.6(200)	49.2(501)
Parental supervision			
No	36.7 (537)	36.6 (286)	36.7 (251)
Yes	63.3 (923)	63.4 (496)	63.3 (425)
Bullied			
No	47.7 (610)	44.7 (306)	51.1 (304)
Yes	52.3 (652)	55.3 (369)	48.9 (283)
Seriously considered suicide			
No	78.0(1066)	78.7 (539)	76.5 (501)
Yes	22.0 (300)	21.3 (146)	23.5 (154)

Note: Missing values were not included in the calculation of percentages

Table 2 indicates that male subjects were less likely to contemplate committing suicide than females (OR = 0.71; 95% CI [0.55, 0.93]). Feelings of loneliness were positively associated with suicide ideation for both males and females (OR = 1.60; 95% CI [1.03, 2.33] and OR = 2.09; 95% CI [1.43, 3.03]). Worry and alcohol drinking were positively associated with suicide ideation among females only (OR = 2.42; 95% CI [1.66, 3.54] and OR = 1.81; 95% [1.05, 3.13]). For both males and females, being bullied was positively associated with suicide ideation (OR = 1.62; 95% CI [1.06, 2.47] and OR = 1.63; 95% CI [1.08, 2.46]).

**Table 2:** Bivariate Logistic regression results between having considered suicide and predictor variables

	Total	Males	Females
Age (years)			
11–13	1.00	1.00	1.00
14	1.12 [0.68, 1.86]	1.51 [0.64, 3.59]	0.96 [0.17, 1.81]
15	1.23 [0.77, 1.98]	1.14 [0.51, 2.53]	1.43 [0.48, 1.75]
16–17	1.23 [0.78, 1.95]	1.77 [0.82, 3.79]	0.96 [0.52, 1.76]
Gender			
Females	1.00	-	-
Males	0.71 [0.55, 0.93]	-	-
Worry			
No	1.00	1.00	1.00
Yes	1.71 [1.31, 2.22]	1.25 [0.86, 1.82]	2.42 [1.66, 3.54]
Loneliness			
No	1.00	1.00	1.00
Yes	1.79 [1.37, 2.32]	1.60 [1.03, 2.33]	2.09 [1.43, 3.04]
Cigarette smoking			
No	1.00	1.00	1.00
Yes	1.75 [1.01, 3.04]	1.59 [0.81, 3.12]	2.35 [0.74, 7.45]
Drinking			
No	1.00	1.00	1.00
Yes	1.40 [0.99, 2.00]	1.12 [0.68, 1.85]	1.81 [1.05, 3.13]
Parental supervision			
No	1.00	1.00	1.00
Yes	0.87 [0.66, 1.13]	0.93 [0.63, 1.36]	0.81 [0.55, 1.18]
Bullied			
No	1.00	1.00	1.00
Yes	1.58 [1.18, 2.11]	1.62 [1.06, 2.47]	1.63 [1.08, 2.46]

Table 3 presents results from multivariate analysis. Worry and loneliness remained positively associated with suicide ideation after adjusting for age, gender, cigarettes smoking, alcohol drinking and experience of having been bullied (OR = 1.19; 95% CI [1.12, 2.25] and OR = 1.59; 95% CI [1.12, 2.26]). Likewise, male gender remained negatively associated with suicide ideation in multivariate analysis (OR = 0.70; 95% CI [0.50, 0.98]).

**Table 3:** Multivariate Logistic regression results between having considered suicide and predictor variables

Age (years)	
11–13	1.00
14	0.91 [0.49, 1.70]
15	0.90 [0.50, 1.63]
16–17	1.15 [0.66, 2.00]
Gender	
Females	1.00
Males	0.70 [0.50, 0.98]
Loneliness	
No	1.00
Yes	1.59 [1.12, 2.26]
Worry	
No	1.00
Yes	1.19 [1.12, 2.25]
Cigarette smoking	
No	1.00
Yes	1.29 [0.55, 3.01]
Drinking	
No	1.00
Yes	1.27 [0.80, 2.01]
Bullied	
No	1.00
Yes	1.34 [0.96, 1.87]

*Adjustments made for age, gender, loneliness, worry and cigarette smoking.

## Discussion

21.6 percent of the study participants, 21.3% males and 23.5% females had seriously considered committing suicide within the past 12 months. Factors positively associated with a history of suicidal ideation were female gender, being victim of bullying, lack of parental supervision, current cigarette smoking, and alcohol use. All of these factors have been described as being associated with suicidal ideation in other studies [8-12]. Thirty-six percent of the general population has reported history of suicidal ideation Uganda [13]. The estimate from Uganda is much lower than the 39.7% life time prevalence among Viennese students [5]. However our study looked at 12 months period and so comparisons between the two settings may not be reasonable. Due to the design of our study, we are unable to ascribe causation i.e. it is not possible to say these factors are causes of suicidal ideation.

Over half (52.3%) of the study participants in rural Uganda reported being victims of bullying. This prevalence estimate is much higher than 19.3% found among school going adolescents in Durban and Cape Town in South Africa [14]. In the Uganda study, participants were asked the question: During the past 30 days, on how many days were you bullied? In the South African study, students were asked: During the past 12 months, have you been bullied at school? Even though the Uganda study asked for a much shorter period (30 days compared to 12 months in South Africa), the Uganda survey was associated with a much higher estimate. We are unable to specifically identify the reasons for these differences. A possibility is that indeed adolescents in rural Uganda are more likely to be bullied than in South Africa. The second possibility is the way the questions were asked. In South Africa, the questions aimed to identify bullying occurring at school. In the Uganda study, this was not specified and study participants may have reported bullying occurring in and out of school. Despite these differences, the prevalence of having been bullied in the past 30 days appears to be unacceptably high in rural Uganda.

Current cigarette smoker (5.7%) was significantly lower than the 21.9% reported by Mpabulungi and Muula for Arua, rural Uganda for 2002 [15]. While in our study cigarette smoking is for a representative sample of all rural Uganda, Mpabulungi and Muula estimates were for one district, which was also tobacco growing. Bolton et al have reported that use of alcohol and cigarette smoking may be initiated or maintained as self-medications among persons who have anxiety disorders, in themselves risk factors for suicide [16]. Liu et al have reported increased prevalence of suicidal ideation with age among youths in rural China [17]. We also found that the odds of suicidal ideation increased with increasing age.

Studies from other settings have reported female over-representation among participants reporting suicidal ideation has been reported elsewhere [8-10]. In our study however, the gender disparity was minimal i.e. 21.3% and 23.5% among males and females.

Females may also be over-represented among individuals who actually attempt or commit suicide [18,19]. In a facility based study of 45 suicide and attempted suicide patients in rural Namibia, Ikealumba and Couper reported that 23 were female while 21 were males [20].

While the gender disparity in suicidal ideation in many settings is that females are more likely to have suicidal thoughts, the gender disparity is reversed with regard to suicide commission i.e. males are more likely to commit suicide and using more violent means than females [21]. Brezo *et al.*[22] have found that female gender was also associated with a 2–3 times greater likelihood of suicide attempts among Canadian-French participants.

## Limitations of this study

Our study has several limitations. We did not collect data on other factors that may have effect on suicidality. For instance evidence reported in a meta-analysis by Bridge et al suggest that antidepressant drugs may be associated with suicidality [23]. Other studies have also demonstrated that psychiatric co-morbidity is an important proximal determinant of suicidal behaviors [24-26]. These other factors were not assessed largely because we used secondary data. The study also recruited participants from school-going adolescents. To the extent that school-going adolescents are different in their prevalence and factors associated with suicidal ideation, our study may not be representative of all adolescents in rural Uganda. It is also important to recognize that not all suicidal ideation will result in suicide attempt or suicide [21]. However, adolescents who have suicidal thoughts are more likely to attempt or commit suicidal compared to those without the thoughts. Brezo et al have reported that correlates of suicide attempts in suicidal ideators may vary as a function of the persistence of suicidal ideas and gender [22]. Among Canadian-French study participants, these authors found that persistent suicidal ideas, Axis I psychopathology, female gender and childhood sexual abuse (CSA) were the most consistent correlates of suicide attempts. Externalizing disorders were more important among males.

## Conclusion

Our study has found that the prevalence of suicidal ideation among adolescent students in rural Uganda was 21.6%. Being females, increasing age, cigarette smoking, alcohol, having been bullied, loneliness, significant worry and lack of parental supervision were associated with suicidal ideation. There is need to recognize suicidal ideation as an important issue among adolescents in rural Uganda. The design, implementation and evaluation of public health interventions aimed at reducing suicide in Uganda should incorporate our understanding of the factors associated with suicide. Ovuga *et al.*[27] have described how mental health care leaders in Uganda are currently promoting the provision of care where for decades since independence, mental health has been largely neglected. This initiative has potential to mobilize attention and support towards mental health research and care in Uganda.

## Abbreviations

CDC: Centers for Disease Control and Prevention

GSHS: Global School-Based Health Survey

UNAIDS: Joint United Nations Program on HIV/AIDS

UNESCO: United Nations Educational, Scientific and Cultural Organization

UNICEF: United Nations Children's Fund

WHO: World Health Organization

## Competing interests

The author(s) declare that they have no competing interests.

## Authors' contributions

ER conducted the data analysis and participated in drafting of manuscript.

ASM conceived the analysis plan, participated in interpretation of data and drafting of manuscript.

SS participated in the interpretation and drafting of manuscript.

JT coordinated in-country data collection, participated in the interpretation of findings and drafting of manuscript.

All authors approved the final draft of the manuscript.

## Pre-publication history

The pre-publication history for this paper can be accessed here:


